# Signaling and crosstalk of rhizobacterial and plant hormones that mediate abiotic stress tolerance in plants

**DOI:** 10.3389/fmicb.2023.1171104

**Published:** 2023-06-30

**Authors:** B. N. Aloo, J. Dessureault-Rompré, V. Tripathi, B. O. Nyongesa, B. A. Were

**Affiliations:** ^1^Department of Biological Sciences, University of Eldoret, Eldoret, Kenya; ^2^Department of Soil and Agri-Food Engineering, Laval University, Quebec, QC, Canada; ^3^Department of Biotechnology, Graphic Era (Deemed to be University), Dehradun, Uttarakhand, India

**Keywords:** plant growth promoting rhizobacteria, phytohormones, abiotic stress tolerance, water stress, drought stress, temperature stress

## Abstract

Agricultural areas exhibiting numerous abiotic stressors, such as elevated water stress, temperatures, and salinity, have grown as a result of climate change. As such, abiotic stresses are some of the most pressing issues in contemporary agricultural production. Understanding plant responses to abiotic stressors is important for global food security, climate change adaptation, and improving crop resilience for sustainable agriculture, Over the decades, explorations have been made concerning plant tolerance to these environmental stresses. Plant growth-promoting rhizobacteria (PGPR) and their phytohormones are some of the players involved in developing resistance to abiotic stress in plants. Several studies have investigated the part of phytohormones in the ability of plants to withstand and adapt to non-living environmental factors, but very few have focused on rhizobacterial hormonal signaling and crosstalk that mediate abiotic stress tolerance in plants. The main objective of this review is to evaluate the functions of PGPR phytohormones in plant abiotic stress tolerance and outline the current research on rhizobacterial hormonal communication and crosstalk that govern plant abiotic stress responses. The review also includes the gene networks and regulation under diverse abiotic stressors. The review is important for understanding plant responses to abiotic stresses using PGPR phytohormones and hormonal signaling. It is envisaged that PGPR offer a useful approach to increasing plant tolerance to various abiotic stresses. However, further studies can reveal the unclear patterns of hormonal interactions between plants and rhizobacteria that mediate abiotic stress tolerance.

## 1. Introduction

Abiotic stressors such as drought, salt, cold, and heat are key limiting variables in modern agriculture and account for more than 50% of plant outputs. These pressures restrict plant development by affecting several physiological processes, including photosynthesis, pigment content, and water relations (Pathak et al., 2014). For instance, cell elongation of plants can be inhibited by the interruption of water flow from the xylem to the surrounding cells (Kaamali et al., 2019). In beans, Mathobo et al. (2017) showed that genotypes had reduced grain yield, as well as chlorophyll levels, and photosynthesis and transpiration under water stress. The effects of salt stress on the physiological traits of wheat (Saddiq et al., 2020), rice (Mekawy et al., 2015; Razzaq et al., 2020), and other plant genotypes have also been established. Drought, heat, and salt stresses are especially important because of the production of reactive oxygen species (ROS) that affect plant metabolism (Hasanuzzaman et al., 2020, 2021). The interactions between plants and soil can also shape the root microbiome under abiotic stress and the general impacts of these abiotic stresses on crops (Koevoets et al., 2016; Hartman and Tringe, 2019; Chai and Schachtman, 2022; Duan et al., 2022). Cognizant of this, the major concern is to enhance crop productivity for food security by increasing crop tolerance to various abiotic stresses.

Plant growth regulators or hormones are chemical compounds that dramatically affect the development and differentiation of plant cells, tissues, and organs and serve as intercellular chemical messengers. Now, it is obvious that these hormones influence plant stress tolerance by modifying plant physiology and/or gene expression patterns. Several studies have investigated and documented the ability of plant growth regulators and phytohormones to improve plant resistance to abiotic stress (Shu et al., 2018; Santhi et al., 2021). To protect themselves, plants employ a variety of biochemical pathways, which stimulate a collection of phytohormones, growth regulators, and signaling molecules. This is the interaction or cross-talk between these events and processes which helps in plant protection (Roychoudhury and Aftab, 2021). Nonetheless, the majority of research has evaluated the phytohormones generated by plants to mediate their tolerance to abiotic stress. It is now evident that the plant rhizomicrobiome, including plant growth-promoting rhizobacteria (PGPR), also generates phytohormones that govern plant development (Kalimuthu et al., 2019). PGPR have been studied for decades (Kloepper et al., 1980), and their numerous mechanisms of action have been divided into direct mechanisms such as nitrogen fixation, phosphate solubilization, siderophore production, and phytohormones production. Indirect mechanisms such as antibiosis, induced systemic resistance, competition for nutrients, parasitism and production of diverse metabolites (Upadhyay et al., 2016) also exist and both direct and indirect mechanisms have been related to promoting plant growth. There have also been efforts over the years to determine the function of rhizobacterial phytohormones in plant tolerance to drought, water, and salt stressors, among others (discussed in Section 4). Such microbiomes are essential for building and sustaining plant resilience to various abiotic stressors.

Hormones exert their effects by activating the production of other hormones or the expression of genes. These transduction cascades regulate gene expression, which directly influences the production or function of several hormones and developmental processes (Khan et al., 2020). Further identification and investigation of these compounds and the crosstalk between them and plant-produced phytohormones that mitigate the negative effect of environmental stress in plants can shed significant light on the hormonal signaling and crosstalk that can be exploited to improve plant tolerance to abiotic stresses. Increased resistance to abiotic stresses such as heat, drought, and acidity is required more than ever as a result of climate change which is anticipated to increase the plant abiotic stressors (Teshome et al., 2020). The objective of this review is to evaluate the functions of PGPR phytohormones in plant abiotic stress tolerance and outline the current research on rhizobacterial hormonal communication and crosstalk that govern plant abiotic stress responses. This review first presents the principal forms of abiotic plant stressors (Section 2), and the functions of rhizobacteria and their phytohormones in plant abiotic stress tolerance in Sections 3 and 4. The review then recalls the rhizobacterial hormonal signaling crosstalk that mediates abiotic stress tolerance (Section 5) and highlights the new elements and ideas on the same (Section 6).

## 2. Major types of plant abiotic stresses

**Figure 1:**
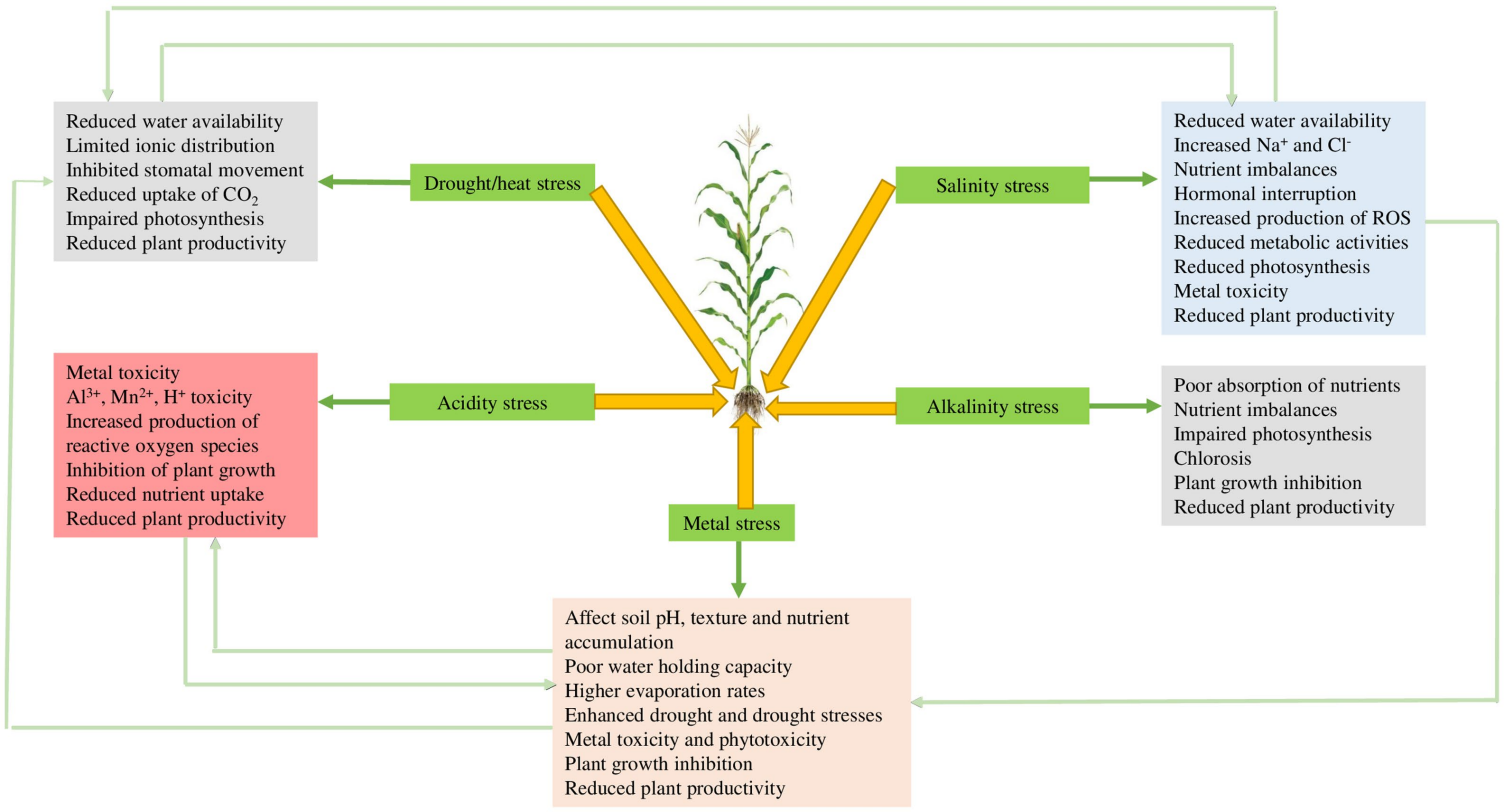
Summary of abiotic stresses in plants.

Plants are very sensitive to drought, heat, temperature, cold, salinity, heavy metals, and other abiotic stresses. It is estimated that abiotic stresses may account for more than 50% of crop yield loss depending on crop type and magnitude of exposure (Kajla et al., 2015). Here we discuss the different types of abiotic stresses and their effects on plant growth. Brief overviews of the main types of plant abiotic stresses are presented in sub-sections 2.1 to 2.5. The effects of several abiotic stresses in plants are summarized in Table 1 and a schematic illustration of the same is shown in Figure 1. Figure 1 shows some important abiotic stress including drought/heat, salinity, pH-related stress, and metal stress. Each of these abiotic stress impacts normal plant functioning and stimulates different types of responses to alleviate the stress. Sections 2.1 to 2.5 detail these different plant abiotic stresses and their effects on plant growth and physiology.

**Table 1:** Effects of abiotic stresses on plant physiology, growth, and development.

Abiotic stress	Plant	Effects	Test conditions	Country of study	References
Acidity	Wheat (*T. aestivum*)	Affect lipid peroxidation and antioxidative capacity	Greenhouse	South Africa	Tóth et al. (2020)
	Tea (*Camellia sinensis*)	Growth and development of plants were hampered by metabolic problems brought on by increased acidity, which also hindered photosynthesis and the antioxidant defense system	Field	China	Zhang et al. (2020)
Drought	Wheat (*T. aestivum*)	Decreased grain weight	Potted/growth chamber	China	Li et al. (2018)
	Rapeseed (*Brassica naous*)	Alteration of various physiological and anatomical parameters	Potted/growth chamber		Zhu et al. (2021)
	Cowpeas (*Vigna unguiculata*)	Reduced fresh weights of the leaves and the whole plant, oxidized soluble sugars and lipids in the leaves and roots, and increased electrolyte leakage from the leaves	*In vitro*	Canada	Jayawardhane et al. (2022)
	Cassava (*Manihot esculenta*)	Plant height, stem diameter, leaf number, leaf water content, net photosynthetic rate, intercellular CO_2_ concentration, and stomatal conductance were all lowered	Greenhouse	China	Shan et al. (2018)
	*Chrysanthemum indicum*	Reduced shoot and root lengths, number of flowers, photosynthetic activity, stomatal conductance	Potted	India	Sahithi et al. (2021)
	Sesame (*Sesamum indicum*)	Germination percentage, coefficient of variation in germination time, germination index, and seedling vigor index all decreased	Growth chamber	Pakistan	Ahmed et al. (2022)
	Tomatoes (*Solanum lycopersicum*)	Oxidative stress and the production of antioxidants	Potted	China	Zhou et al. (2019)
Salinity	Wheat (*T. aestivum*)	Lower growth and water content relative. Leaf withering and curling is an early sign of leaf senescence	*In vitro*/potted	Pakistan, China	Ilyas et al. (2020) and Li et al. (2018)
	Tomato (*S. lycopersicum*)	When the amount of salt in the germinating medium increased, the percentage of seeds that germinated dropped	*In vitro*	Bangladesh	Chakma et al. (2019)
	Chickpea (*Cicer arietinum*)	Plant growth, plant height, dry biomass, and yields are reduced by 15%–32%	Greenhouse	Australia	Atieno et al. (2017)
	Sorghum (*Sorghum bicolor*)	A lower germination rate, a higher germination index, longer seedling shoots and roots, as well as fresh and dry weight	*In vitro*	Iran	Dehnavi et al. (2020)
	Rice (*Oryza sativa*)	Sluggish germination, reduced shoot and root dry weight, shoot and root length, and fresh weight	*In vitro*	India	Vibhuti et al. (2015)
Temperature	Tomato (*S. lycopersicum*)	Production of reactive oxygen species in like superoxide and hydrogen peroxide	Greenhouse	Korea	Muneer et al. (2016)
	Rice (*O. sativa*)	Delayed germination, dry weight of shoot and root, shoot and root length, fresh weight of stem and root decreased	*In vitro*	India	Vibhuti et al. (2015)
Water	Cowpeas (*V. unguiculata*)	A reduction in the amount of soluble sugar in leaves, an increase in the amount of lipid peroxidation in the leaves and roots, and an increase in the amount of leaf electrolyte leakage were all observed	*In vitro*	Canada	Jayawardhane et al. (2022)
	Maize (*Zea mays*), Sorghum (*S. bicolor*)	Plant height and stem diameter were significantly reduced by salt and water stress	Field	Sudan	Mohammed and Mohammed (2019)

### 2.1. Drought and heat

Drought is the most severe form of abiotic plant stresses globally and is expected to be more severe in the coming decades due to climate change. The severity and global impact of drought stress are unparalleled relative to other environmental stresses. Drought stress can especially have devastating effects on plant growth since 80–95% of fresh plant biomass is composed of water which is necessary for a multitude of physiological and biochemical processes. Roots are usually the first plant organs to perceive and respond to drought stress. However, the effects of drought stress are apparent throughout the plant to hinder plant function and productivity (Heckathorn et al., 2013). For instance, drought-induced reduced water availability in the rhizosphere limits ionic distribution, turgor pressure, and homeostasis in plant cells, thereby inhibiting nutrient uptake by roots. Short-term consequences for plant growth include decreased carbon assimilation, stomatal closure, osmotic adjustment, inhibition of growth, hydraulic changes, signal transport, and cell-drought signaling (Ahluwalia et al., 2021) Interference with homeostasis can also affect stomatal functions and limit the uptake of CO_2_ for photosynthesis (Shan et al., 2018; Talbi et al., 2020). Heat stress can be short-term (often called heat-shock) or longer-term (often called heat-wave) and both stress induces strong negative impacts during various stages of plant growth (Jagadish et al., 2021). For example in wheat Akter and Islam (Akter and Islam, 2017) observed that heat stress significantly reduces seed germination and seedling growth, cell turgidity, and plant water-use efficiency, generating excessive reactive oxygen species (ROS), leading to oxidative stress. In addition, wheat encountered leaf senescence, reduction of photosynthesis, deactivation of photosynthetic enzymes, and generation of oxidative damages to the chloroplasts, leading to reduces grain number and size. According to Hussain et al. (2019), the overproduction of ROS such as superoxide (O_2_) and hydrogen peroxide (H_2_O_2_) may be caused by drought and heat stress that denature proteins and peroxidize lipids to impair membrane structure. Among the proteins that can be denatured by ROS under drought stress are chlorophylls (Amiri et al., 2018). Thus, drought and heat stress can hamper photosynthetic activities and crop productivity in the long run (Choukri et al., 2020; Sahithi et al., 2021). The general symptoms of drought and heat-stressed plants include leaf rolling, yellowing, scorching, plant stunting, and permanent wilting (Żurek et al., 2018). Drought stress can also affect important plant functions and interactions like the rhizosphere microbiomes and root exudation patterns as discussed in Chen et al. (2022).

### 2.2. Salinity

Salinity and drought stresses are closely related in terms of occurrence and consequences on plant growth. As salt levels in soils become more saline, less than optimal water becomes available to plant roots. Nevertheless, plants only become stressed when the concentration of Na^+^ and Cl^−^ which consequently alters several metabolic activities in them elevates in soil beyond the thresholds for normal functioning. Due to the high salt concentration in soil, plants can suffer from hyperosmotic and hyper-ionic effects (Hasegawa et al., 2000).

Salt stress may increase the outflow of electrolytes from plant cells by dislodging Ca from membranes, hence reducing membrane permeability and causing a greater efflux of electrolytes (Hniličková et al., 2019). This has recently been confirmed by Ilyas et al. (2020) who, while researching the rhizobacteria from salty soils that generate salt tolerance in wheat, noticed greater electrolyte leakage in wheat (*Triticum aestivum*) at 150 mM salt stress compared to the control. Typically, these pressures cause membrane disorganization, decreased metabolic activity, altered ion, and mineral absorption rates, and reduced water intake. Salinity-induced imbalances in water and nutrient uptake eventually significant losses in plant productivity and yield. Salinity can also affect plant productivity by restricting leaf growth with marked effects on photosynthesis (Chen et al., 2019; Kwon et al., 2019). Besides, plants subjected to salinity show enhanced production of ROS which damages DNA, proteins, and membranes (Saini et al., 2018; Al Kharusi et al., 2019). Salinity also increases heavy metal toxicity as secondary stress (Zhou et al., 2019). More discussions on metal stress in plants are in Section 2.4.

### 2.3. Acidity

Soil acidity is a substantial worldwide limitation on agricultural output. The United States Department of Agriculture’s Natural Resources Conservation Service defines acidic soil as having a pH of 6.5 or below (Burt, 2014). Since it affects almost every step of the nutrient absorption process, soil pH has a substantial impact on plants. Al^3+^, Mn^2+,^ and H^+^ are the three main toxicities that acidic soils expose plants to, and they all prevent plant development. For instance, aluminum toxicity prevents roots from growing, dividing, and absorbing nutrients (Bojórquez-Quintal et al., 2017). The amount of ROS, such as O_2_ and H_2_O_2_, which oxidize essential cellular components, may be affected in acid soils. For instance, in acidic circumstances, rice seedlings have been shown to have enhanced lipid peroxidation and H_2_O_2_ content (Zhang et al., 2015). Lipid peroxidation has also been reported in acidic conditions in *Plantago* (Martins et al., 2013).

### 2.4. Alkalinity

High levels of bicarbonates (HCO_3_^−^) and carbonates (CO_3_^2−^) are present in alkaline soils as a result of weathering and dryness, which prevent sufficient water from being available to drain soluble salts from the soil (Rashid et al., 2019). Crops are equally impacted by alkalinity, partly because the ROS brought on by both stressors increases plant oxidative damage (Bui et al., 2014; Fatima et al., 2021). Alkalinity and salinity can co-occur in some soils with more detrimental effects on plant growth, nutrient uptake, and root morphology as has been shown in *Lotus tenuis* (Paz et al., 2012). The combined consequences of alkalinity and salinity, however, are often more severe than either alkalinity or salinity alone (Shi and Sheng, 2005).

Alkaline soils can limit the bioavailability of phosphorus (P), iron (Fe), and zinc, which causes nutritional imbalances in plants (Zn). Alkalinity may also limit photosynthesis because HCO_3_^−^decreases iron absorption, which therefore affects how chlorophyll is made (Bayarash et al., 2020). As a result of Fe shortage, Fe-induced chlorosis predominates in calcareous soils. Due to the high concentrations of HCO3-in the soil that prevents root respiration, alkalinity may also impede root development. This is most likely the cause of the stunted development seen in most alkaline-stressed plants (Singh et al., 2018).

### 2.5. Heavy metals

Heavy metals are often defined as metals with specific weights of more than 5 g/cm^3^. Lead (Pb), nickel (Ni), cadmium (Cd), aluminium (Al), manganese (Mn), and iron (Fe) among others. These heavy metals may be produced by the soil’s natural weathering processes (Wu et al., 2020; Dinter et al., 2021), the use of metal-based pesticides and fertilizers (Chen et al., 2020), or volcanic eruptions. The majority of soil physio-chemical characteristics, such as pH, soil texture, and the accumulation of nutrients, are impacted by metal toxicity, which has an impact on total plant development (Jia et al., 2020). Metal-contaminated soils impact negatively plant development, root growth, photosynthetic activity, and therefore, mineral nutrient accumulation (Yang et al., 2020). Heavy metal stress damages enzymes and other important proteins through inactivation or denaturation. Heavy metals disrupt the substitution reaction of essential metallic ions with biomolecules, which interferes with membrane integrity and diminishes photosynthetic capacity, respiration, and other cellular processes (Hossain et al., 2012). Heavy stress also triggers the generation of hydrogen peroxide, superoxide radicals, and hydroxyl radicals causing oxidative stress (Hossain et al., 2012; Ghori et al., 2019). Cadmium, for example, may produce phytotoxicity and oxidative stress in other sections of the plant, which can lower plant productivity (Tran and Popova, 2013; Sabir et al., 2020). Inadequate development and morpho-physiological harm may also result from higher Pb concentrations in plant cells (Salam et al., 2019).

## 3. Rhizobacterial mediation of abiotic stress tolerance in plants

Rhizobacteria are a group of microorganisms that can improve plant growth and health via nutrient solubilization, nitrogen fixation, and the synthesis of phytohormones, siderophores, and pathogen-repelling biomolecules (Ahemad and Kibret, 2014). The importance of PGPR in reducing abiotic stress has grown significantly over time. Several studies on rhizobacteria help plants adapt to abiotic stressors like acidity (Zhang et al., 2020), drought (Kang et al., 2014; Manjunatha et al., 2022), salinity (Islam et al., 2016; Zerrouk et al., 2019; Siddiqui et al., 2022), and heavy metals (Mesa-Marin et al., 2018; Kazerooni et al., 2021).

**Figure 2:**
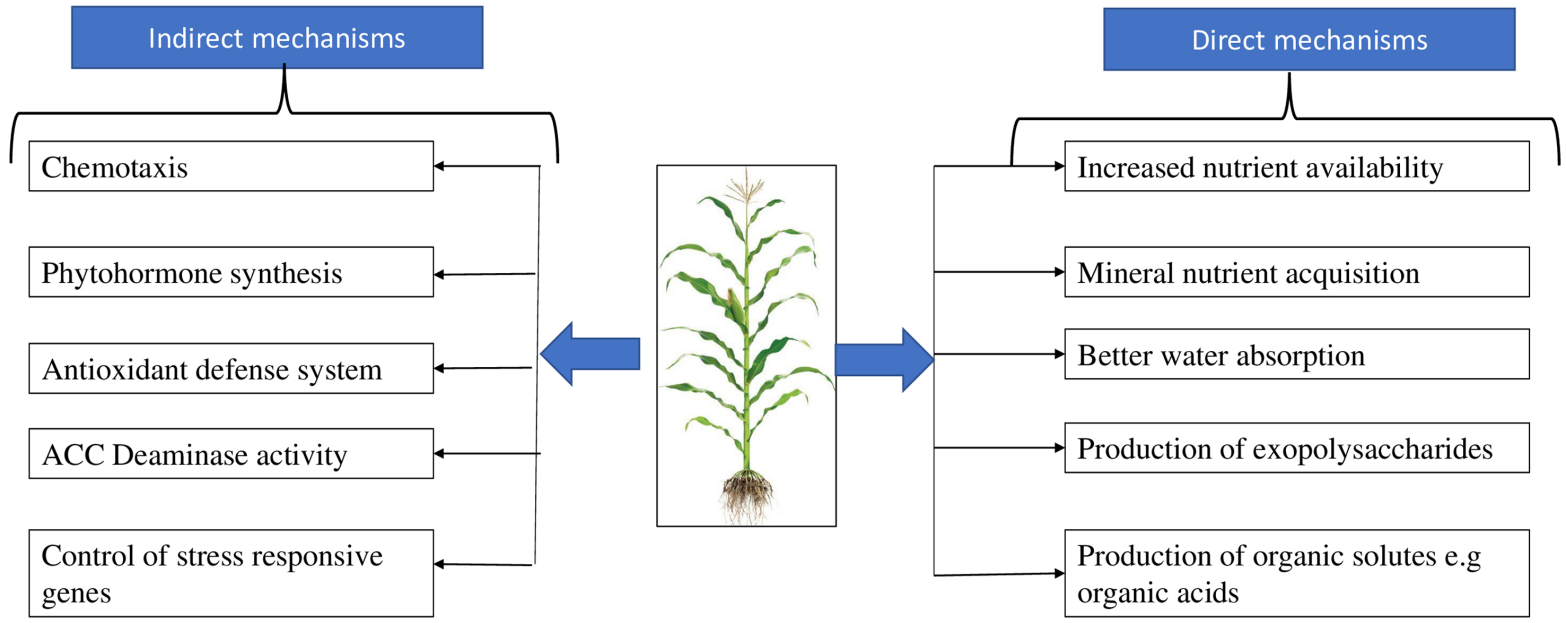
Direct and indirect actions of plant growth promoting rhizobacteria that promote plant abiotic stress tolerance.

Plant stress is lessened by PGPR through direct and indirect processes (Figure 2). Aspects of the direct action of PGPR against plant abiotic stresses include increased nutrient availability, mineral acquisition, better water absorption, and development of exopolysaccharides, biofilm, and many organic solutes, including sugars, organic acids, amino acids, and polyamines (Goswami and Suresh, 2020; Kazerooni et al., 2021). As for the indirect mechanisms through which plant stress can be alleviated by PGPR they include: chemotaxis, phytohormone synthesis and level variations, antioxidant defense system activation, 1-aminocyclopropane-1-carboxylate (ACC) deaminase activity, and the control of stress-responsive genes in plants all play a role in indirect action of PGPR against plant abiotic stresses (Rosier et al., 2018). These activities control multiple biological processes in plants and together contribute to local and distant rhizobacterial-induced tolerance to abiotic stresses in plants.

Many rhizobacteria that improve or modulate plant tolerance to abiotic stresses have been shown to produce various phytohormones that support plants under these conditions (Hassan and Ahmed, 2018; Ilyas et al., 2020). Phytohormones-mediated systemic plant tolerance to abiotic stresses is achieved through various physical and chemical changes and regulation of genetic and signaling networks affecting the growth and development processes.

Rhizobacteria can also reduce heavy-metal-induced plant stress by detoxifying, biosorption, bioaccumulation in cells, or bioleaching (Huang et al., 2016; Kumar et al., 2020). They release siderophores, which are low-molecular-weight chelators that combine with metals such as Cd, Fe, Pb, and Zn to form complexes (Rajkumar et al., 2009, 2010; Ma et al., 2011). The production of siderophores by rhizosphere microbes has recently been investigated for the mitigation of stress in plants (Singh et al., 2022). This phytohormone-producing and heavy-metal-tolerant PGPR may be utilized to detoxify heavy metal-polluted soils effectively, affordably, and sustainably, even though under heavy metal stress, microorganisms may encounter a significant degree of metal toxicity (Kamnev et al., 2005). Studies that demonstrate the critical roles of rhizobacteria phytohormones in the alleviation of various abiotic stresses in plants are further discussed in Section 4.

## 4. Rhizobacterial and plant hormones as signaling molecules that modulate abiotic stress tolerance in plants

**Figure 3:**
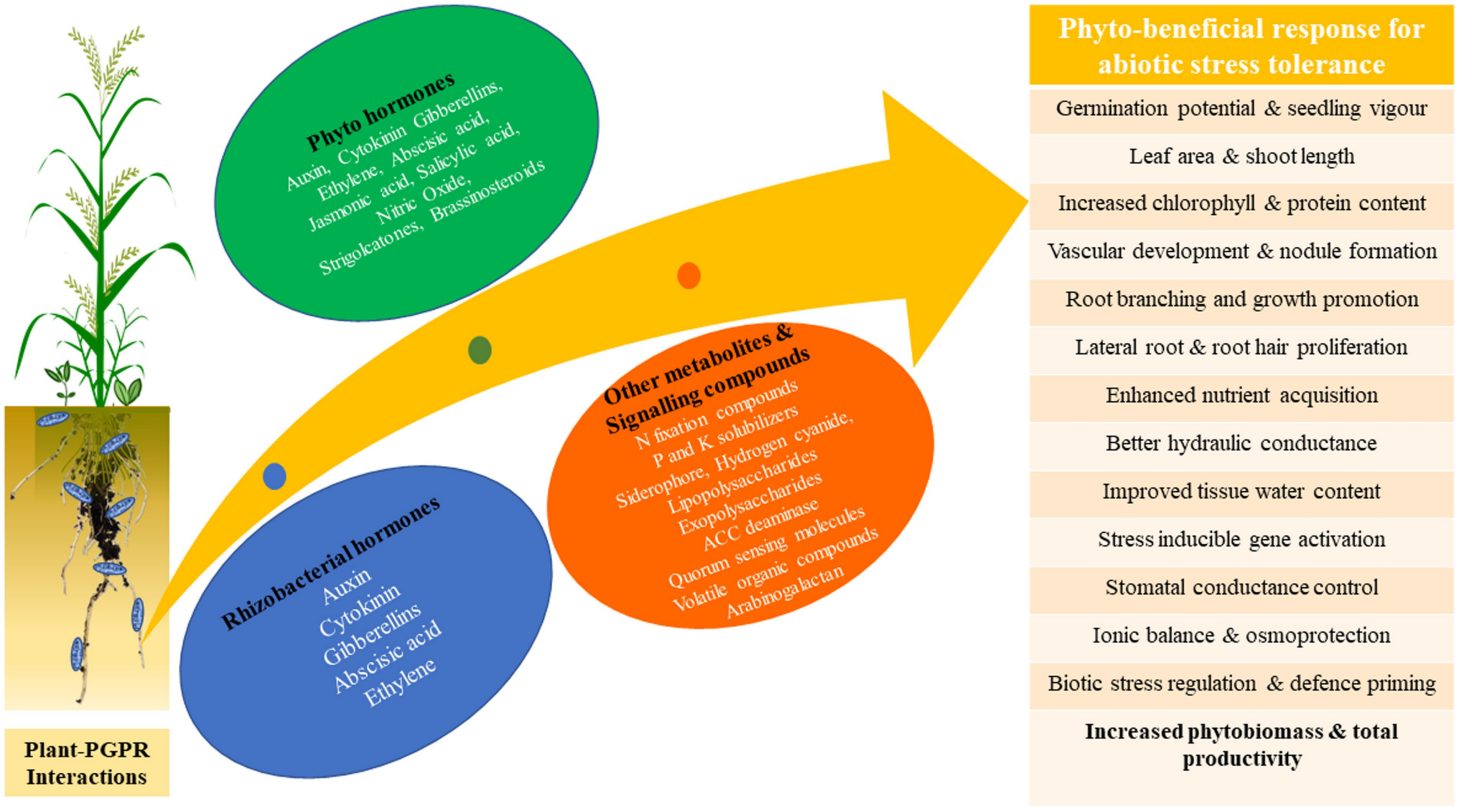
Roles of plant and rhizobacterial phytohormones in plant abiotic stress tolerance.

**Figure 4:**
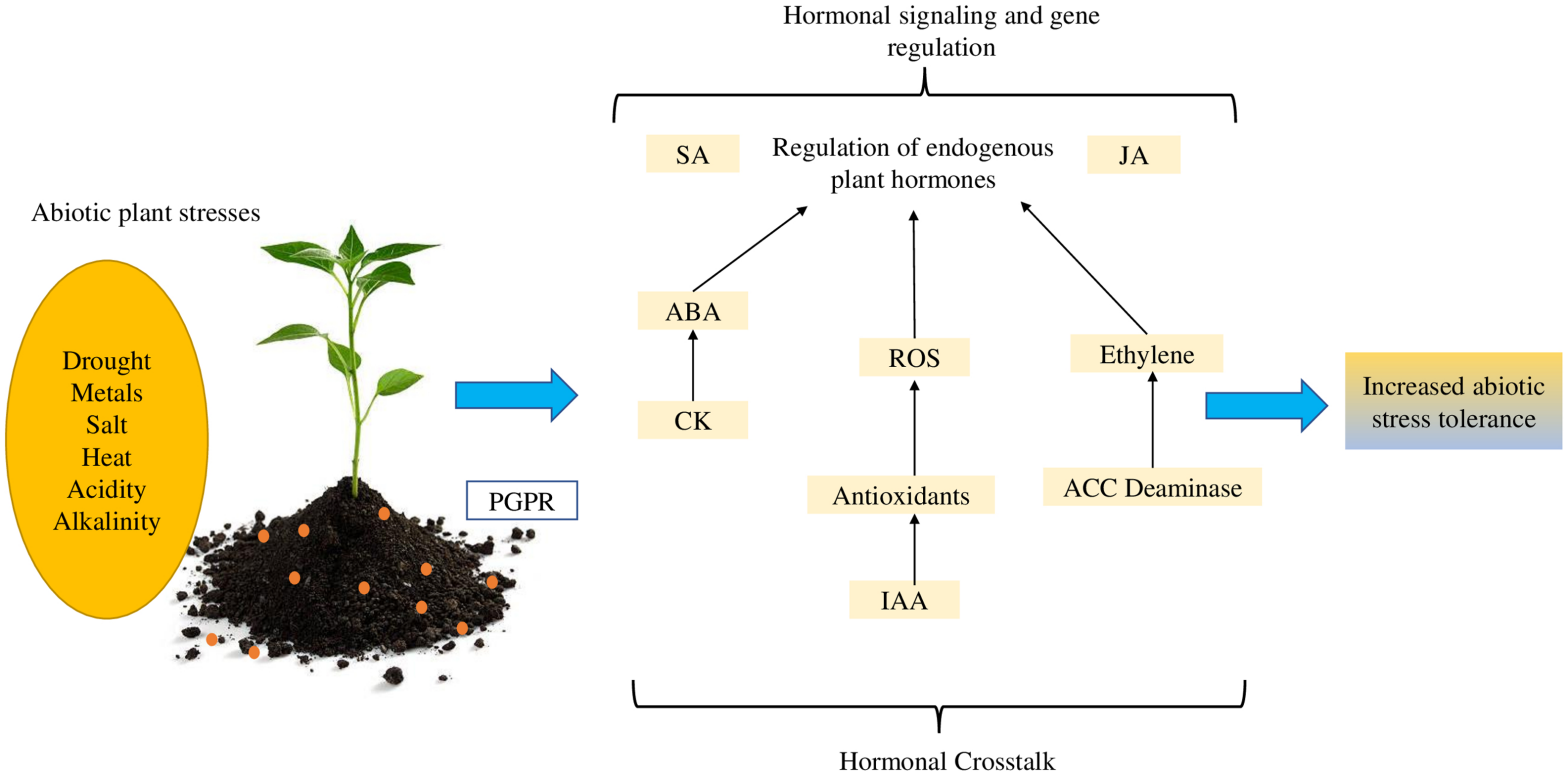
Rhizobacterial hormonal signaling and crosstalk with plant-endogenic phytohormones that mediate abiotic stress tolerance in plants.

Phytohormones of plant and rhizobacterial origin are important bioregulators for a variety of cellular functions and signaling pathways in plants. These hormones include auxins (AUX), cytokinins (CK), abscisic acid (ABA), ethylene (ET), salicylic acid (SA), jasmonic acid (JA), gibberellic acids (GA), strigolactones (SL), and brassinosteroids (BRs) (Vocciante et al., 2022). According to reports, each phytohormone has a role in several cellular and biological processes that influence plant growth and development. Although PGPR only produces extremely little levels of phytohormones, they are nonetheless essential for plant development, including the control of abiotic stress tolerance (Table 2). We discuss the major phytohormones that contribute to plant abiotic stress tolerance in sub-Sections 4.1 to 4.7. Figure 3 summarizes the functions of plant and rhizobacterial phytohormones in plant abiotic stress tolerance (see Figure 4).

**Table 2:** Rhizobacterial phytohormones that mediate abiotic stress tolerance in plants.

Abiotic stress	Rhizobacteria	Phytohormones	Crop	Test conditions	References
Drought/heat	*Azospirillum brasilense*	ABA, ET	Maize (*Z. mays*)	Potted	Curá et al. (2017)
	*Bacillus tequilemsis*	GA, IAA	Soybean (*Glycine max*)	Potted	Kang et al. (2019)
	*B. subtilis*	CK	*P. orientalis*	Potted	Liu et al. (2013)
	*Methylobacterium oryzae*	CK	Lentils (*Lens culinaris*)	Growth Chamber	Jorge et al. (2019)
	*P. fluorescens*	CK	Tomato (*Solanum lycopersocum*)	Potted	Mekureyaw et al. (2022)
	*P. fluorescens*	IAA	Wheat (*T. aestivum*)	Potted	Chandra et al. (2018)
	*Bacillus* spp.	IAA, GA	Maize (*Z. mays*)	Potted	Vardharajula et al. (2011)
	*Bacillus* spp., *Enterobacter* spp	IAA	Velvet bean (*M. pruriens*)	Potted	Saleem et al. (2018)
	*Pseudomonas aeruginosa*	IAA	Mung bean (*Vigna radiata*)	Lab, pot, field	Uzma et al. (2022)
	*Ochrobactrum pseudogrignonense*, *Pseudomonas* sp., *B. subtilis*	IAA	Black gram (*Vigna mungo*)	*In vitro*/potted	Saikia et al. (2018)
	*Achromobacter xylosoxidans*, *Pseudomonas oryzihabitans*, *Variovorax paradoxus*	Auxins	Potato (*Solanum tubrosum*)	Potted	Belimov et al. (2015)
	*A. baldaniorum*	SA, JA, ABA	Purple basil (*Ocimum basilicum*)	Hydroponic system	Mariotti et al. (2021)
	*B. aryabhattai*	IAA, JA, GA	Soybean (*G. max*)	Potted	Park et al. (2017)
Salinity	*A. woluwensis*, *M. oxydans*, *A. aurescens*, *B. megaterium*, *B. aryabhattai*	IAA, GA	Soybean (*G. max*)	*In vitro*	Khan et al. (2019)
	*Glutamicibacter arilaitensis*, *Enterobacter cloacae*, *Leclercia adecarboxylata*, *B. subtilis*,	IAA	Tomato (*S. lycopersicum*)	Potted	Haque et al. (2022)
	*Pseudomonas pseudoalcaligenes*, *B. subtilis*	IAA	Soybean (*G. max*)	Hydroponic system	Yasmin et al. (2020)
	*Aneurinibacillus aneurinilyticus*, *Paenibacillus*	IAA	French bean (*Phaseolus vulgaris*)	Potted	Gupta and Pandey (2019)
	*Bacillus xiamenensis*	IAA	Sugarcane (*Saccharum officinarum*)	Greenhouse/potted	Sharma et al. (2021)
	*Pseudomonas stutzeri*, *Klebsiella pneumoniae*	IAA	Rice (*O. sativa*)	Greenhouse/potted	Khumairah et al. (2019)
	*Pseudomonas plecoglossicida*	IAA	Maize (*Z. mays*)	Greenhouse/potted	Zerrouk et al. (2019)
	*Glutamicibacter* sp.	IAA	Rice (*O. sativa*)	*In vitro*	Ji et al. (2020)
	*Rhizobium*	IAA	Canola (*Brassica napus*)	Potted	Saghafi et al. (2018)
	*P. putida*	IAA	Citrus (*Citrus latifolia*)	Potted	Vives-Peris et al. (2018)
	*Ancylostoma braziliense*, *Azotobacter chroococcum*	IAA, CK	*Coriander* spp.	Potted	Rabiei et al. (2020)
Salinity & alkalinity	*Bacillus licheniformis*	ABA	*Chrysanthemum* spp.	Potted	Zhou et al. (2017)

ABA, abscisic acid; ET, ethylene; GA, gibberellic acids; CK, cytokinins; IAA, indole-3-acetic acid; SA, salicylic acid; JA, jasmonic acid.

### 4.1. Indole-3-acetic acid

One of the most prevalent phytohormones, indole-3-acetic acid (IAA), promotes root nodulation, cell proliferation, and differentiation to increase the root surface area available for the absorption of nutrients and water (Zhang et al., 2005). While IAA and other AUX are among the most researched phytohormones, we still know very little about how they could modulate plant development in response to stress. Rhizobacteria may also synthesize the hormone, albeit in small quantities, and increase plant tolerance to abiotic stressors (Table 2).

The inoculation of plants with IAA-producing rhizobacteria has also been associated with improved root volume and length and better absorption of nutrients and water under drought stress (Manjunatha et al., 2022). The production of IAA by rhizobacteria has recently been related to reduced ET levels in plants (Barnawal et al., 2017; Saleem et al., 2018). While studying the drought response of Velvet beans (*Mucuna pruriens*), Saleem (Saleem et al., 2018) found considerably lower ET levels in the roots and shoots of plants inoculated with IAA-producing rhizobacteria compared to un-inoculated controls. Ethylene is a stress hormone that controls plant senescence and changes or restricts plant development in response to abiotic stress (see Section 4.6). The inoculation of water-stressed purple basil with IAA-producing *Azospirillum baldaniorum* also improved the leaf water status (Mariotti et al., 2021). These and other results summarized in Table 2 demonstrate that IAA-producing rhizobacteria are ubiquitous and enhance plant growth under abiotic stress.

Several routes for IAA production in plants and microorganisms have been discovered through genetic analysis. The indole-3-pyruvate route, which produces IAA most often, relies on the indole-3-pyruvate decarboxylase enzyme, which is encoded by the ipdC gene. The process of turning indole-3-pyruvate into indole-3-acetaldehyde is mediated by the enzyme indole-3-pyruvate decarboxylase (Patten and Glick, 2002; Baudoin et al., 2010). An alternate mechanism comprises the decarboxylation of tryptophan into indole-3-acetamide by tryptophan monooxygenase (iaaM) and the hydrolysis of indole-3-acetamide into indole acetamide by an indole acetamide hydrolase (iaaH) (Spaepen et al., 2007). Indirect-based mechanisms such as the production of siderophore are also associated with IAA production by *Bacillus subtilis* which in turn improved sesame plant growth and oil content (Nithyapriya et al., 2021).

### 4.2. Cytokinins

Cytokinins control plant growth and development via cell division, seed germination, apical dominance, delayed leaf senescence, and chlorophyll buildup in leaves (Zwack and Rashotte, 2013, 2015). The most prevalent cytokinin in plants is zeatin, which may exist in both trans and cis forms. Trans zeatin is an active cytokinin in all plant species, but cis-zeatin is active in just a subset of plant species and is ubiquitously present in select plant species (Gajdošová et al., 2011). In plants, adenosine phosphate-isopentenyl transferases (IPTs) and CK oxidases/dehydrogenases control CK metabolism (CKXs). Whereas the former is encoded by IPT genes and is responsible for the production of CK, the latter is often involved in the degradation of CK by cleaving its side chains and causing its reversible inactivation (Mok and Mok, 2001).

Although plants generally respond to drought by reducing their CK levels, a PGPR-induced increase in CK levels may help plants to tolerate drought. Recent research has shown that *Azospirillum brasilense* enhances wheat development under water deficit (Zaheer et al., 2022). Similarly, CK-producing *Pseudomonas fluorescens* has recently been shown to improve the growth of tomatoes under water deficit (Mekureyaw et al., 2022). A strain of *Sinorhizobium meliloti* was engineered by Xu et al. (2012) to overproduce CK and then tested for the ability to protect alfalfa crops against drought stress (Xu et al., 2012). After a period of severe drought stress, alfalfa plants inoculated with the bacteria producing higher CK were significantly bigger than plants that were inoculated with the non-transformed strain. This experiment demonstrated how CK can boost the tolerance of alfalfa (and possibly other crops) to severe drought stress (Xu et al., 2012). However, there is limited research on the role of CK-producing rhizobacteria in mediating plant tolerance to abiotic stresses. Cognizant of this, there is a need for more research since the mechanisms that confer tolerance to one abiotic stress involving CK may or may not apply to other types of abiotic stressors.

### 4.3. Gibberellic acids

Gibberellic acids constitute a class of active or inactive tetracyclic diterpenoid carboxylic acids involved in a variety of plant developmental processes (Bao et al., 2020). Two soluble oxoglutarate-dependent dioxygenases catalyze the divergence of GA12 into two branches in the cytosol, one of which leads to the creation of GA4 and the other to GA1 and GA3 (Hedden and Thomas, 2012). In response to abiotic stress, GA-mediated signaling demonstrates crosstalk with other phytohormones such as ABA and IAA, therefore integrating numerous hormonal signaling pathways.

As with other hormones, GA-producing PGPR may control the endogenous GA content of host plants to compensate for the decreased synthesis of the hormone by stressed plants. For example, GA-producing bacteria promote the growth and development of many crops in salty environments (Kang et al., 2014). Under abiotic stress, gibberellic acids produced by PGPR are also responsible for leaf expansion and stem elongation. *Bacillus aryabhattai* generated several forms of GA *in vitro* and boosted the development of heat-stressed soybean plants (Park et al., 2017). *Arthrobacter woluwensis*, *Microbacterium oxydans*, *Arthrobacter aurescens*, *Bacillus megaterium*, and *B. aryabhattai* generate GA that promotes soybean growth in salt conditions (Kang et al., 2019). Nevertheless, the role of GA-producing bacteria in reducing the impacts of drought on host plants remains largely understood.

### 4.4. Abscisic acid

Abscisic acid is the most common stress hormone in plants with classical roles in many osmotic responses under salt and drought stress. It controls most plant-water relations under drought stress, for example, in maize (Zhang et al., 2012). its synthesis is stimulated by water-limiting conditions in both plants and PGPR. Under water-limiting conditions, PGPR and plant-produced ABA control stomatal closure to decrease transpiration rates, and regulate dehydration by stress signal transduction (Asghari et al., 2020). Stomatal closure is the most typical ABA response to drought stress through the limitation of water loss. ABA production increases in the roots of drought-stressed plants, from where it is transported to the shoots to trigger stomatal closure (Verma et al., 2016). This way, PGPR inoculation can increase drought tolerance by increasing ABA accumulation in plants, which leads to stomatal closure and decreased rates of leaf transpiration. In rice (*Oryza sativa*), inoculation with ABA-producing *B. amyloliquefaciens* has recently been shown to increase salinity tolerance, confirming that phytohormone production by PGPR is responsible for plant resistance to antibiotics (Shahzad et al., 2017). Hence, ABA and/or its analog produced by rhizobacteria may control phytohormonal status, boost plant growth, and regulate plant responses to drought stress. The production of ABA is controlled by 9-cis epoxycarotenoid dioxygenase; whose expression is proportional to endogenous ABA levels (Thompson et al., 2000). The effect of *Azospirillum lipoferum* in maize plants treated with ABA and GA inhibitor showed that *Azospirrillum*-inoculated plants better-alleviated water stress. Among the mechanisms involved in water stress alleviation by plants by *Azospirillum* is the production of stress-type hormones such as ABA together with auxins and GA (Cohen et al., 2009).

### 4.5. Salicylic and jasmonic acids

Salicylic acid is a phenolic molecule that enhances photosynthetic and growth characteristics in plants and protects against oxidative damage caused by abiotic stressors (Wani et al., 2017). During drought stress, it works as a signaling molecule, activating genes that encode heat-shock proteins, chaperones, and antioxidants. Moreover, jasmonic acid participates in the activation of the antioxidant system and the control of stomatal opening (Yang et al., 2019).

Similarly, to other hormones, bacterial SA synthesis may have a protective function in abiotic stress tolerance by adding to the pool of endogenously generated plant SA and its signaling pathway. Yet, there is minimal evidence that bacterial SA directly contributes to abiotic tolerance. Recently, *A. baldaniorum*-treated purple basil plants recorded increased SA and JA contents in the xylem sap which improved water stress tolerance in purple basil (Mariotti et al., 2021). *Bacillus aryabhattai*-treated soybean plants have also shown higher levels of JA than uninoculated plants and produced longer roots and shoots compared to those of control plants (Park et al., 2017). Similarly, *B. amyloliquefaciens* increased JA contents in *Glycyrrhiza uralensis* roots under drought stress (Yue et al., 2022). These reports provide evidence that PGPR may function in the modulation of plant JA and SA contents by activating the biosynthesis pathways involved.

### 4.6. Ethylene

Ethylene plays a crucial role in plant response to abiotic stressors in addition to governing essential plant activities such as blooming, seed germination, and senescence. For example, under drought circumstances, ET decreased stomatal conductance, which reduced intercellular CO_2_ concentration and lowered photosynthetic (Pierik et al., 2006). During plant ET biosynthesis, methionine is first transformed to S-adenosyl methionine and then ACC is formed. Drought-induced stimulation of root ET biosynthesis results in increased ET biosynthesis by the ACC-oxidase enzyme in plants. This is a typical response to the stimulation of root ET biosynthesis. Moreover, ethylene interacts with other hormones, such as auxins and ABA, to regulate the plant’s reaction to a lack of water. In Section 5 of this article, these interactions are examined in further detail. Yet, under stress, excessive ET synthesis might inhibit root growth and plant development. By breaking down the ET precursor ACC into alpha-ketobutyrate and ammonia, the production of the enzyme ACC-deaminase by PGPR serves to control ET biosynthesis during drought stress (Van de Poel and Van Der Straeten, 2014). This has previously been demonstrated in red pepper (*Capsicum annuum*) by Siddikee et al. (2011). Overall, this facilitates the survival of plants by lowering the ET levels.

## 5. Rhizobacterial hormonal signaling and crosstalk with plant hormones that mediate abiotic stress tolerance in plants

It is now obvious that both plants and PGPR create phytohormones that, among other crucial activities, influence plant tolerance to abiotic stressors. Yet, via influencing plant hormones and physiological responses, the PGPR may also regulate abiotic stress tolerance in plants. There are reports on rhizobacterial hormonal signaling and interplay with phytohormones that mediate abiotic stress tolerance in plants (Defez et al., 2017; Kang et al., 2019). It is widely known that crosstalk between ABA, ET, SA, and JA with AUX and GA affects gene networks controlling stress tolerance in plants (Khan et al., 2020). According to Poupin et al. (2016), Many hormonal signaling pathways in plants are carefully controlled by PGPR strains, hence influencing several physiological functions. For instance, crosstalk with other phytohormones improves plant salt stress resistance as reviewed by Egamberdieva et al. (2017).

Hormonal cross-talk facilitates the organization of several genes and their regulators in response to stress. Similar to phytohormones that are administered exogenously, microbe-produced phytohormones alter the hormone levels in plants. This may occur when microorganisms change the hormonal balance of the plant by secreting growth regulators or promoting their production inside the plant (Cabot et al., 2014). According to Barnawal et al. (2017) and Sorty et al. (2016), many PGPR enhance plant tolerance to water stress by secreting phytohormones and modifying plant endogenous hormone levels. This has recently been confirmed by Manjunatha et al. (2022) who investigated the effects of two PGPRs on pearl millet under drought stress and established higher contents of plant-produced ABA, GA, and IAA in inoculated plants than in the controls. The inoculants increased the drought resistance of the plants by modifying the hormones, physiology, and gene expression.

Modifications in endogenous IAA levels in plants owing to inoculation with IAA-secreting PGPR result in enhanced drought resistance have been previously reviewed by Dodd et al. (2010). Yet, there is no proof that the higher IAA level in these plants is a result of the PGPRs’ absorption of IAA. It is hypothesized that a greater accumulation of IAA in the shoot and root of the inoculated plant, whether stressed or not, indicates either the uptake of IAA secreted by *Pseudomonas putida* by the plants or the stimulation of a signaling cascade by bacterial IAA to upregulate endogenous IAA biosynthesis in *Arabidopsis* (Ghosh et al., 2019). Strong cross-talk between ABA and CK is one of the most important components of drought stress detection in plants since increased ABA accumulation inhibits CK production. Recent results of decreased concentrations of endogenous CK and elevated levels of ABA in water-stressed non-inoculated plants support this (Ghosh et al., 2019).

Several PGPR produce hormones that augment the plant-derived hormones to improve the abiotic stress tolerance of plants. The PGPR may increase ABA or GA production and control plant hormone levels (Dodd et al., 2010). In investigating CK-producing, PGPR-mediated drought resistance in *Platycladus orientalis* seedlings, we observed CK-producing, PGPR-mediated drought resistance. Previous studies by Liu et al. (2013) have confirmed that CK and ABA produced by *Bacillus subtilis* increased drought tolerance in *P. orientalis*. In a second investigation, *A. brasilense* inoculation of potatoes in planta affected the hormonal system by raising the levels of IAA and CK in the stems and leaves. The researchers determined that the bacteria produce this hormone for absorption by the plants (Arkhipova et al., 2020).

The modulation of plant hormonal and stress physiology under abiotic stresses has been demonstrated by several studies. For instance, *P. putida* inoculation under drought stress has also been shown to reduce this gap by 10.22% and the concentration of ABA was greater in inoculated plants than in untreated ones (Kang et al., 2014). The addition of *P. putida* to drought-and salt-stressed plants mitigated the negative effects of the stressors by lowering ABA levels. Another stress hormone, JA, was reduced in *P. putida*-inoculated plants relative to their control counterparts. Similar amounts of JA were detected in salt-stressed and drought-stressed plants, respectively. The interaction between *P. putida* and drought-and salt-stressed plants resulted in a considerable increase in JA concentration. Furthermore, abiotic stressors and bacterial contact also affected SA concentration in inoculated plants relative to other treatments, and the control, plants subjected to salt stress followed by drought stress had a considerably higher SA concentration. *P. putida* improved soybean development under drought or salt stress by raising or reducing the levels of endogenous SA (Kang et al., 2014). It has been postulated that the exogenous application of beneficial bacterial cultures may offset the inhibitory effects of various abiotic stressors by modulating SA biosynthesis. The stress resistance given by PGPRs may be due to the modulation of phytohormones such as ABA, JA, and SA, which correlate to plant defense and growth (Kang et al., 2014; Carvalhais et al., 2015, 2017). In Kang’s (Kang et al., 2019) study, in heat-stressed soybean plants inoculated with *B. tequilensis*, the endogenous levels of JA and SA were dramatically elevated, but the endogenous level of ABA was significantly downregulated. In contrast, the stress-responsive hormone ABA content of soybean plants was considerably downregulated by PGPR-inoculation in this research, suggesting that PGPR lowered the amount of stress in the plants. These results suggest a potential cross-talk between auxin and SA, which modulates plant tolerance responses.

## 6. Emerging areas and perspectives

Overall, it is well-established that PGPR-produced phytohormones alongside those of plant origin help plants to withstand abiotic stresses by improving the upregulation of antioxidant systems, enhancing photosynthetic potential, regulating the stomatal conductance, and stimulating root development for increased uptake of water and mineral nutrients (Khan et al., 2020). However, it is still not fully understood how the PGPR-produced phytohormones coordinate cellular activities and regulate the cellular process in abiotic stress responses. Many current studies are underway that will further define the role of rhizobacterial hormones in PGPR-primed abiotic stress tolerance.

Fluctuations in temperature and precipitation are likely to increase in a changing global climate (Intergovernmental Panel on Climate Change, 2013). Since most abiotic stresses occur together, specific plant responses mediated by rhizobacterial hormonal signaling for plants under combined stresses cannot be predicted from the results of studies based on single abiotic stresses. Hence, it is increasingly becoming important to assess rhizobacterial hormonal signaling for plant tolerance to multiple abiotic stresses which is normally the scenario with climate change. Most studies using rhizobacterial isolates for plant abiotic stress tolerance have dwelt on isolates from abiotically-stressed environments for plant inoculation and testing. This is especially very promising since such PGPR isolated can have a stronger potential to mitigate similar stresses in plants due to their natural adaptive mechanisms to specific stress conditions. Therefore, they have likely developed mechanisms to tolerate and mitigate the adverse effects of abiotic stresses. By inoculating plants with such PGPR, the plants can benefit from the inherent stress tolerance mechanisms possessed by these strains. Plant inoculation with such PGPR inoculation may improve crop growth and yield under a variety of abiotic situations. These indigenous PGPR strains are well suited to local conditions, but they must be studied and documented before they can be used as crop inoculants.

Moreover, soil-plant-microbe interactions should be studied to determine the effectiveness of these microorganisms with varying physicochemical properties under various abiotic stresses. Specialist research is also required to explore the mechanisms that lead to the synthesis of different metabolites, as well as their synergistic and antagonistic interactions with host plants. Genetic studies can also discover the best time to locate receptors for the synthesis of certain genes in host plants after microbial inoculation, as well as the genetic modifications of related bacteria. Plant-microbe interactions should be optimized using molecular genetics, bioinformatics, and modeling tools to maximize agricultural productivity and soil and environmental health. Such new frontiers have been evidenced by some studies (Prabhavathi et al., 2002; Abd El-Daim et al., 2018; Jha and Subramanian, 2018). There are, however, some challenges regarding designing experiments that accurately mimic the complex and dynamic conditions of natural ecosystems. Abiotic stresses, such as drought, salinity, or temperature extremes, vary in intensity, duration, and frequency in different environments. Therefore, replicating these conditions in controlled settings while maintaining experimental rigor can be difficult. Abiotic stresses can also vary spatially and temporally within a given ecosystem. Thus, accounting for this variability in experimental designs and interpreting results across different stress conditions can be complex. Similarly, abiotic stress responses in plants and microbes often involve dynamic physiological and molecular changes over time. Understanding the temporal dynamics of soil-plant-microbe interactions under abiotic stresses requires long-term monitoring, which can be resource-intensive. Maintaining experimental conditions over extended periods and capturing the full range of temporal responses is challenging.

Advances in technology and genetics, such as the use of transcriptomics to identify the transcript components involved in the early stages of rhizobacteria-induced systemic tolerance to drought stress, may provide light on the molecular mechanism behind the signal transmission. Many studies have shown that phytohormone-secreting PGPR has a good influence on the general health, physiological condition, and endogenous levels of a few hormones in plants under drought stress. Little is known, however, about how a particular strain of PGPR might assist plants to cope with drought stress by altering the accumulation and localization patterns of the four primary endogenous hormones. It is unclear if these changes are the consequence of bacterial phytohormone absorption by plants, a change in the plant’s endogenous hormone metabolism driven by bacteria, or a combination of the two. Moreover, there is a lack of thorough knowledge of how PGPR controls endogenous plant hormones.

## 7. Conclusion

Rhizobacterial phytohormones are key macromolecules that influence several physiological processes in plants. Despite advancements in understanding the physiological and molecular processes of plant abiotic stress tolerance, the particular mechanisms of rhizobacteria-mediated plant tolerance to abiotic stresses remain unexplained. Nevertheless, an increased understanding of rhizobacterial hormones and hormonal signaling in improving crop growth and yield under multiple abiotic stresses might provide an alternative strategy for developing environmentally friendly and sustainable agricultural practices, especially in the wake of climate change which brings about multiple stresses to plants. Hormonal crosstalk is pervasive and occurs in many forms. To deconstruct the multilayered responses under abiotic stress conditions, a considerable understanding of plant responses to combined stress exposure is also required. The present review has deconstructed signaling and crosstalk of rhizobacterial and plant hormones that mediate abiotic stress tolerance in plants. This discovery opens up new opportunities for the investigation of beneficial interactions in plants. A deeper understanding of root-microorganism consortiums might also lead to new research avenues for vital agricultural areas whose sustainability depends on these interactions.

## Author contributions

All authors listed have made a substantial, direct, and intellectual contribution to the work and approved it for publication.

## Funding

The publication of this manuscript was supported with funds from Laval University, Quebec-Canada.

## Publisher’s note

All claims expressed in this article are solely those of the authors and do not necessarily represent those of their affiliated organizations, or those of the publisher, the editors and the reviewers. Any product that may be evaluated in this article, or claim that may be made by its manufacturer, is not guaranteed or endorsed by the publisher.

## Conflict of interest

The authors declare that the research was conducted in the absence of any commercial or financial relationships that could be construed as a potential conflict of interest.
